# Alterations in the prevalence and serotypes of *Streptococcus pneumoniae* in elderly patients with community-acquired pneumonia: a meta-analysis and systematic review

**DOI:** 10.1186/s41479-025-00156-0

**Published:** 2025-02-25

**Authors:** Xinyue Luo, Qianli Yuan, Jing Li, Jiang Wu, Binghua Zhu, Min Lv

**Affiliations:** 1 The 305 Hospital of PLA, Beijing, 100017 China; 2 Beijing Center for Disease Prevention and Control, Beijing, 100013 China

**Keywords:** Community-acquired pneumonia, *Streptococcus pneumoniae*, Elderly, Meta-analysis

## Abstract

**Background:**

Pneumococcal pneumonia is a common disease with a significant impact on morbidity and mortality among the elderly population. The main purpose of this meta-analysis was to estimate the prevalence of community-acquired pneumonia (CAP) in elderly individuals caused by *Streptococcus pneumoniae* (*S. pneumoniae*).

**Methods:**

A systematic search of the PubMed, Web of Science, and Scopus databases was conducted for relevant studies published between January 2013 and December 2023. Subgroup analysis and meta-regression were used to identify the sources of heterogeneity affecting the 87,430 patient studies obtained from 47 papers that met the inclusion and exclusion criteria.

**Results:**

The combined prevalence rate for *S. pneumoniae* among all CAP patients included in the study was 14.8% (95% confidence interval [CI]: 12.3–17.8%). The 5-year pooled prevalence decreased from 16.5% (95% CI: 15.0–18.2%) in 1996–2000 to 8.4% (95% CI: 6.3–11.0%) in 2016–2020 for bacterial culture alone and from 17.4% (95% CI: 16.3–18.7%) to 13.5% (95% CI: 10.7–16.8%) for bacterial culture and urinary antigen testing (UAT) combined (*P* < 0.001). The most prevalent serotype was serotype 3, followed by serotypes 8, 19 A, 22 F, 11 A, 5, 9 N, 12 F, 6 A, and 10 A. The vaccine-serotype coverage was 53.5% for PCV 13, 60.5% for PCV 15, 85.2% for PCV 20 and 88.6% for PPSV 23.

**Conclusion:**

These findings indicate a decrease in the overall burden of pneumococcal CAP among elderly individuals over the decade, which lends support to the proposition that the delivery of immunization should be expanded across the life course.

## Introduction

 The increasing prominence of population ageing is a social concern and a major health issue of this era. Community-acquired pneumonia (CAP), especially bacterial CAP, is the primary cause of morbidity and mortality in elderly individuals [1, 2]. Evidence suggests that *Streptococcus pneumoniae* (*S. pneumoniae*) is also considered one of the most clinically significant pathogens mediating pulmonary infections [3]. Pneumococcal disease increases morbidity and mortality substantially in high-risk populations and represents a significant economic and public health burden. 

Five pneumococcal vaccines have been developed and made available worldwide: the 23-valent polysaccharide vaccine (PPSV 23), the 13-valent conjugate vaccine (PCV 13), the 10-valent conjugate vaccine (PCV 10), the 15-valent conjugate vaccine (PCV 15), and the 20-valent conjugate vaccine (PCV 20). New vaccinations that cover a greater number of serotypes are currently undergoing advanced clinical studies. However, the vaccination coverage of the elderly population remains low. In addition, the protection against pneumococcal pneumonia in adults is dependent on the group immunity formed after vaccination in children. Increasing vaccination coverage among the elderly is crucial for mitigating the morbidity and mortality associated with CAP caused by *S. pneumoniae*. Vaccination practices have demonstrated that raising public awareness about the epidemiological profile of the disease can result in increased vaccination rates. However, there is a need to further delineate the burden of laboratory-confirmed pneumococcal disease in the elderly population. To achieve this objective, access to reliable epidemiological data at the country or regional level, as well as more comprehensive and representative data, is essential.

Notably, there is a considerable degree of variation in the prevalence of *S. pneumoniae*, as reported in the published literature for the adult population, with figures ranging from 11.9–68.3% [4]. This discrepancy impairs our comprehension of the role played by *S. pneumoniae* in the pathogenesis of community-acquired pneumonia in elderly individuals. Previous reviews on pneumococcal pneumonia have employed either qualitative studies or early quantitative studies [5–11], of which only one meta-analysis was conducted among adults 14 years ago [6]. The prevalence of pneumococcal pneumonia may have changed over time and is influenced by factors such as vaccination programmes and advances in diagnostic techniques.

The aim of this study was to examine the prevalence of *S. pneumoniae* in elderly patients with CAP in the context of the implementation of pneumococcal vaccination programmes and the diversification of pneumococcal testing methods. We conducted a systematic review and meta-analysis of studies to summarize the prevalence of *S. pneumoniae* in elderly patients with CAP and to explore changes in pneumococcal serotypes. These findings will contribute to the ongoing development of vaccines and provide insights into future prevention strategies.

## Method

### Search strategy

This systematic review was conducted according to the Preferred Reporting Items for Systematic Reviews and Meta-Analysis (PRISMA) guidelines. Up to December 21, 2023, studies on the prevalence of *S. pneumoniae* in elderly CAP patients were retrieved from the PubMed, Web of Science, and Scopus databases. The detailed terms used were “((*Streptococcus pneumoniae*) OR (pneumococcal infection) OR (*S. pneumoniae*)) AND ((community-acquired pneumonia) OR CAP)”. EndNote was used to merge and collect the literature, which was screened according to the following inclusion and exclusion criteria on the basis of a flow chart. To avoid missing literature, reference lists of obtained papers as well as suggestions from other authors were used to identify additional studies.

### Selection criteria

The inclusion criteria were as follows: (1) observational studies such as cross-sectional studies, cohort studies or surveillance; (2) the results of interest derived from CAP patients with an average age of 60 years or older; (3) the results of interest were the infection rate, detection rate, incidence rate or prevalence of *S. pneumoniae* in CAP patients, or the proportion of *S. pneumoniae* in CAP patients; (4) the research object was humans; (5) the samples could have originated from various parts of the body; (6) the studies published from 2013 to 2023; (7) the samples were collected before the COVID-19 pandemic; and (8) there was a computable rate or 95% CI.

### Exclusion criteria

The exclusion criteria were as follows: (1) non-English literature; (2) non-articles such as reviews, meetings, animal experiments, and newspapers; (3) insufficient baseline information to calculate the rate and 95% CI; (4) duplicate literature or data from the same sample; (5) carriage or colonization of *S. pneumoniae* in the nasopharynx or oropharynx; (6) surveys not aimed at research rates (such as case-control studies); (7) research population consisting mainly of immunocompromised people; (8) research in many countries and regions without independent results; (9) sample size less than 50; (10) no results on the proportion of CAP caused by *S. pneumoniae*; (11) average age of cases less than 60 years; and (12) CAP not confirmed by radiographic imaging.

### Data extraction

Articles were reviewed and screened by two reviewers according to the inclusion and exclusion criteria. Decisions were made by the third author in case of disagreement. All the articles judged to meet the inclusion criteria on the basis of the reviewed abstract and title were retrieved for further evaluation. After the full texts of the retrieved papers were reviewed, only those that met all the inclusion criteria were included in the analysis, and the relevant data were extracted.

The two reviewers independently extracted the number of CAP cases and the number of *S. pneumoniae* strains detected in CAP cases included in the literature analysis. The recorded information included the time of the literature review, type of study, age (mean and standard deviation, if reported, median and quartile or age range), sex distribution, source of patients (such as hospitalization, outpatient, emergency and intensive care unit [ICU]), sampling site, *S. pneumoniae* testing method, immune status, underlying disease, and related vaccine vaccination status.

### Statistical analysis

Microsoft Excel was used for organizing the data, R 4.1.3 was used for meta-analysis, and OriginPro Learning Edition was used for data visualization. A meta-analysis of the proportion of *S. pneumoniae* infections in elderly CAP patients with the corresponding 95% CI [12] was performed for all included individual studies. Given the expected large true variation in prevalence among studies, all results meeting the inclusion and exclusion criteria were pooled approximately using a random-effects method, and subgroup analysis was conducted to identify sources of heterogeneity. The *I²* test assessed the variation in the outcomes of all included studies with respect to the objectives. Subgroup analysis included continent, country, level of development (developed country/developing country [according to internationally recognized economic classification standards] [13, 14]), study design (cohort study/cross-sectional study/surveillance), clinical setting (outpatient or emergency/inpatient/ICU), laboratory diagnosis (only bacterial culture/only urinary antigen testing/bacterial culture and urinary antigen testing/only PCR/mixed [combination of multiple detection methods]), average study duration, and average age of patients. In addition, meta-regressions were performed on these covariates to determine the source of heterogeneity. All *P* values were two tailed, and *P* ≤ 0.05 was the threshold for significance.

## Results

### Search results

**Fig. 1:**
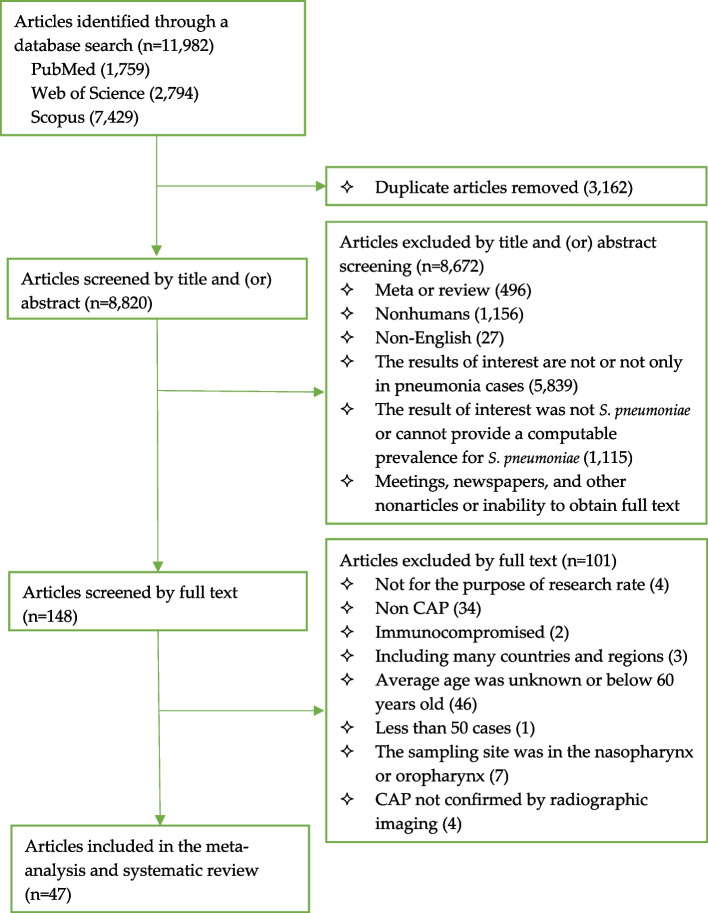
Flow chart for the selection of studies

After the databases were searched, we obtained 11,982 articles, of which 1,759 were from PubMed, 2,794 were from the Web of Science and 7,429 were from Scopus. After duplications were removed, 8,820 articles remained. After the articles were screened by title and/or abstract, we excluded nonhumans; outcomes that we were not interested in; and summary, news or reviews, etc., leaving 148 articles. After the full texts were screened, we further excluded 101 articles according to the inclusion and exclusion criteria. Out of the several thousand initially chosen publications, only 47 articles were ultimately included. The database search is shown in detail in Fig. 1.

### Study characteristics

**Fig. 2:**
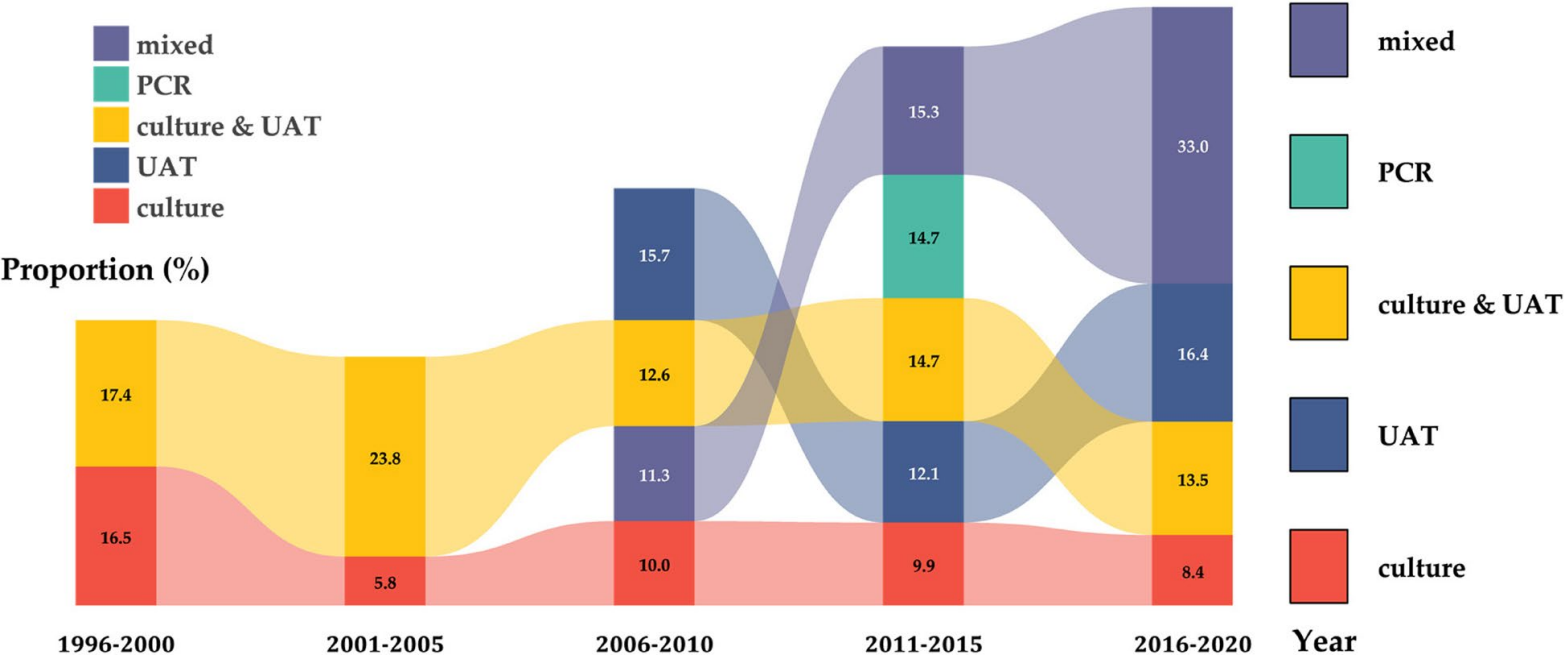
Proportion of CAP caused by *S. pneumoniae* in different studies over time and by type of laboratory diagnosis. Each value represents the pooled percentage of CAP patients in which *S. pneumoniae* was detected over a five-year period, stratified by the initial year of studies. The varying colours indicate the diagnostic methods that were used

The characteristics of the 47 selected studies are presented in Table 1. A total of 87,430 patients were included in the study, with an average age of 68.4 years, and 55.5% were male. The majority of the studies were conducted in Asia, with Japan being the most frequently represented country. The cases were sourced primarily from inpatients in general hospitals (*n* = 59,528; 68.1%), and 6.38% (*n* = 5,576) were drawn from outpatients and emergency departments. The primary samples examined in the study were sputum, blood, and urine. The testing methods included culture, urinary antigen testing, and PCR. Over time, the range of diagnostic methods employed has expanded from a single test to a combination of multiple tests. As the data presented, bacterial culture has been used as a traditional method since 1996. By the year 2000, alternative detection methods with increased sensitivity and specificity had emerged, including urine antigen detection and PCR detection (Fig. 2).

**Table 1:** Basic characteristics of studies included in the review and meta-analysis

First Author (Year)	Study Duration	Region	Average Age (y)	Male (%)	Vaccinated (%)	Source	Setting	Samples	Methods
**Cillóniz, C. (2013)** [15]	1996.11–2008.7	Spain, Europe	78.0	802 (37.3)	310 (14.4)	inpatient	general hospital	BL	culture
**Terraneo, S. (2014)** [16]	2000.1–2011.12	Spain, Europe	64.0	2,529 (62.0)	459 (16.0)	inpatient	general hospital	SP, UR, BAL	culture, UAT
**Ishiguro, T. (2013)** [17]	2002.1–2011.11	Japan, Asia	63.9	683 (66.2)	NS (< 50)	inpatient	general hospital	SP, TS, BAL, BL, PF, UR	culture, UAT
**Khawaja, A. (2013)** [18]	2002.3–2008.12	Pakistan, Asia	68.0	110 (58.0)	NS (< 50)	inpatient	general hospital	SP, TS, BAL, BL	culture
**Spoorenberg, S. M. (2014)** [19]	2004.10–2006.8 2007.11–2010.9	Netherlands, Europe	63.4	295 (58.4)	NS (< 50)	inpatient	general hospital	SP, BL, UR, SR	culture, UAT, ABT
**Molinos, L. (2015)** [20]	2005.11–2007.11	Spain, Europe	66.0	2,859 (65.4)	NS	emergency, inpatient	general hospital	UR, SP, BAL, PF	culture, UAT
**Herrera-Lara, S. (2013)** [21]	2006.1–2009.12	Spain, Europe	63.9	157 (64.6)	NS (< 50)	respiratory unit	general hospital	UR, SP, BL, PF	culture, UAT
**Kumagai, S. (2016)** [22]	2007.4–2014.7	Japan, Asia	76.0	1,073 (69.5)	NS (< 50)	inpatient	general hospital	UR	UAT
**Yoo, K. H. (2013)** [23]	2008.1–2010.12	Korea, Asia	70.1	397 (57.3)	9 (1.3)	respiratory unit	general, referral hospital	SP, UR, PF	culture, ABT
**Holter, J. C. (2015)** [24]	2008.1–2011.1	Norway, Europe	66.0	140 (52.0)	25 (13.0)	inpatient	general hospital	BL, UR	culture, UAT
**Kim, B. (2018)** [25]	2008.4–2017.3	Korea, Asia	65.3	1,417 (62.2)	NS	emergency	general hospital	UR	UAT
**Daniel, P. (2017)** [26]	2008.9–2013.9	England, Europe	71.0	1,223 (55.1)	NS (> 50)	emergency	general hospital	BL, UR	culture, UAT
**Bjarnason, A. (2018)** [27]	2008.12–2009.11	Iceland, Europe	62.8	154 (49.7)	43 (13.8)	inpatient	general hospital	SP, BL, UR	culture, UAT
**Çilli, A. (2018)** [28]	2009.1–2013.9	Turkey, Asia	64.3	417 (67.1)	60 (10.3)	non-ICU	community hospital and general hospital	SP, BL	culture
**Siow, W. T. (2016)** [29]	2009.11–2011.9	Singapore, Asia	61.7	58 (58.0)	NS	ICU	general hospital	SP, BL, UR, TS	culture, UAT
**Gross, A. E. (2014)** [30]	2010.1–2011.12	America, North America	65.0	233 (44.5)	NS (< 50)	inpatient	general hospital	BL, SP, UR, BAL	culture, UAT
**Shindo, Y. (2013)** [31]	2010.3-2010.12	Japan, Asia	75.0	580 (65.4)	NS	inpatient	community hospital and general hospital	SP, TS, BAL, PF, BL	culture
**LeBlanc, J. J. (2017)** [32]	2010.12–2013.12	Canada, North America	68.7	2,553 (53.5)	2,543 (53.3)	internal medicine and ICU	community and nursing hospital	BL, TS, Sterile sites, UR	culture, UAT
**LeBlanc, J. (2020)** [33]	2010–2015	Canada, North America	73.3	3,084 (53.9)	NS (> 50)	internal medicine and ICU	community and nursing hospital	BL, UR	culture, UAT
**LeBlanc, J. J. (2022)** [34]	2010–2017	Canada, North America	72.0	5,846 (52.5)	6,970 (62.6)	inpatient	general hospital	SP, BL, UR	culture, UAT
**Parrott, G. (2017)** [35]	2011.1–2013.12	Japan, Asia	65.0	59 (60.8)	NS	inpatient	general hospital	SP, BL, UR	culture, UAT, SR
**Heo, J. Y. (2018)** [36]	2011.1–2014.12	Korea, Asia	64.0	NS	NS	inpatient, outpatient	general hospital	BL, UR, TS, BAL, SP	culture, UAT
**Iqbal, N. (2020)** [37]	2011.1–2016.12	Pakistan, Asia	63.6	266 (52.6)	NS	inpatient	general hospital	SP, BL, PF, BAL	culture
**Valles, J. (2014)** [38]	2011.4–2012.3	Spain, Europe	60.0	296 (65.9)	NS (< 50)	ICU	general hospital	BL, PF, SP, BAL	culture
**Fukuyama, H. (2013)** [39]	2011.8–2012.7	Japan, Asia	67.9	78 (68.4)	NS (> 50)	inpatient	general hospital	SP, BAL, BL, PF, UR	culture, UAT
**Morimoto, K. (2015)** [40]	2011.9–2013.1	Japan, Asia	77.0	1,040 (59)	356 (35.0)	inpatient, outpatient	community hospital	BL, SP, UR	culture, UAT, PCR
**Torres, A. (2021)** [41]	2011.11–2018.11	Spain, Europe	66.83	1,911 (61.5)	441 (14.2)	inpatient	general hospital	BL, PF, SP, UR	culture, UAT
**Mohamed Faisal, A. H. (2017)** [42]	2011.12–2012.6	Malaysia, Asia	67.0	58 (56.9)	NS	internal medicine and ICU	general hospital	UR, BL, SP, TS, PF, BAL	culture, UAT
**Wu F. (2023)** [43]	2012.3–2018.12	China, Asia	64.0	524 (66.2)	NS	inpatient	general hospital	BAL, TS, SP	PCR
**Yoshii, Y. (2016)** [44]	2012.12–2014.5	Japan, Asia	63.3	59 (64.1)	24 (26.1)	inpatient	general hospital	SP, UR, BL	culture, PCR, UAT
**Mitsui, M. (2021)** [45]	2013–2017	Japan, Asia	76.0	281 (69.7)	NS	inpatient	general hospital	SP, BL, UR, PF	culture, UAT
	2018.7-2018.11		77.0	56 (70.9)					
**Costa, M. I. (2022)** [46]	2013.1–2015.12	Portugal, Europe	71.7	1,058 (55.7)	NS	inpatient	general hospital	BL, SP, UR, PF	culture, UAT
**Pick, H. (2020)** [47]	2013.9–2018.8	England, Europe	71.3	1,515 (51.7)	NS (> 50)	emergency	general hospital	UR, BL	culture, UAT
**Isturiz, R. (2021)** [48]	2013.10–2016.9	America, North America	64.1	5,967 (49.5)	NS	inpatient	nursing hospital	BL, SP, BAL, PF, TS, UR	culture, UAT
**LeBlanc, J. J. (2019)** [49]	2014.1–2015.12	Canada, North America	67.8	3,597 (53.8)	NS (> 50)	internal medicine and ICU	community and nursing hospital	BL, SP, UR	culture, UAT
**Regev-Yochay, G. (2018)** [50]	2014.3–2015.7	Israel, Asia	72.6	310 (62.2)	249 (50.0)	inpatient	general hospital	BL, PF, BAL, TS, UR	culture, UAT
**Lim, Y. K. (2019)** [51]	2015.1–2015.12	Korea, Asia	72.0	121 (51.5)	NS (> 50)	inpatient	referral hospital	UR, BL, PF, BAL, TS, SP	culture, UAT, BacT/Alert 3D blood culture, PCR
**Osman, M. (2021)** [52]	2015.1–2019.12	Thailand, Asia	80.0	251 (51.2)	NS (< 50)	inpatient	general hospital	SP, PF, TS, BL	culture, PCR
**Kara, S. (2019)** [53]	2015.6–2016.12	Turkey, Asia	60.0	87 (65.4)	7 (5.3)	ICU	general hospital	BL, SP, TS, BAL	culture
**Heo, J. Y. (2020)** [54]	2015.9–2017.8	Korea, Asia	64.2	1,500 (56.2)	NS (> 50)	inpatient	hospital and Korean Statistical Information Service database	BL, SP, UR	culture, UAT
**Şenol, E. (2021)** [55]	2016.11–2017.1	Turkey, Asia	69.0	286 (61.5)	15 (3.3)	outpatient, emergency	general hospital	BL, SP, UR	culture, UAT
**Lin, W. H. (2022)** [56]	2016.11–2018.12	China, Asia	67.8	147 (69.3)	NS	outpatient, emergency	general hospital	BL, SP, PF, BAL, TS	culture
**Gilbert, D. N. (2021)** [57]	2017.1–2018.3	America, North America	66.3	148 (54.0)	NS	inpatient	community hospital	SP, TS, BL	culture, UAT
**Liapikou, A. (2022)** [58]	2017.11–2019.4	Greece, Europe	70.6	272 (56.4)	NS	inpatient	community hospital and general hospital	BL, TS, PF, UR	culture, UAT
**Rögnvaldsson, K. G. (2023)** [59]	2018.5–2019.4	Iceland, Europe	75.0	209 (41.1)	NS	inpatient	general hospital	BL, SP, UR	culture, UAT
**Hyun, H. (2022)** [60]	2018.7–2019.6	Korea, Asia	70.0	360 (64.6)	NS (> 50)	inpatient	referral hospital	SP, BL, UR	culture, UAT
**Kawecki, D. (2022)** [61]	2019.1–2020.10	Poland, Europe	70.9	252 (63.3)	NS (> 50)	emergency	general hospital	SP, BL, UR	culture, UAT

*Abbreviations: *SP* sputum, *BL* blood, *UR* urine, *BAL* bronchoalveolar, *PF* pleural fluid, *TS* tracheobronchial secretions, *SR* serum, *UAT* urinary antigen testing, *ABT* antibody testing, *CAP* community-acquired pneumonia, and *NS* no state

### S. pneumoniae

**Fig. 3:**
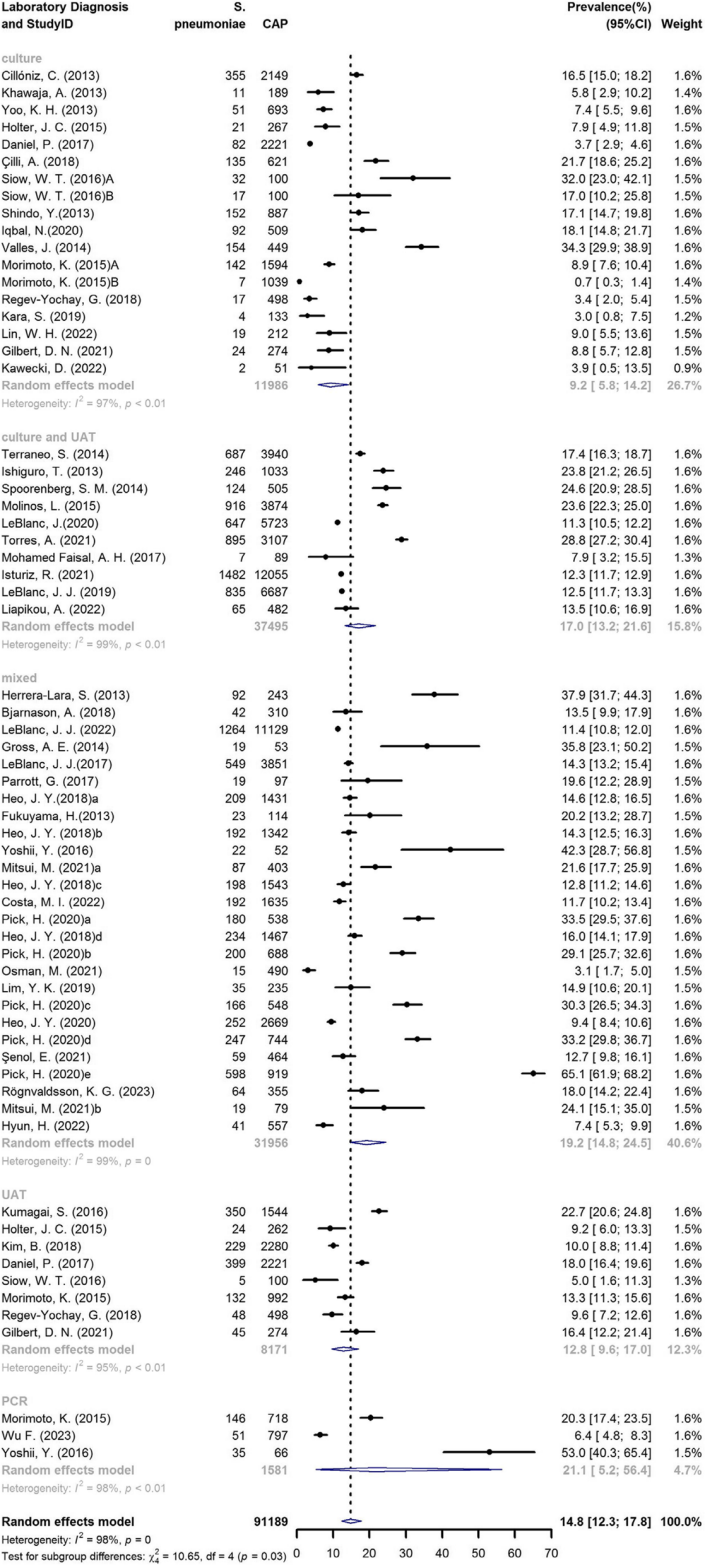
Unadjusted, study-specific proportions of *S. pneumoniae* as the causative agent for CAP, stratified by type of laboratory diagnosis. (The letters a, b, c, d and e represent the results from different research years in a single publication. The letters A and B represent the results from different types of laboratory diagnosis in a single publication. The term *mixed* indicates the combination of multiple detection methods in a single study.)

Among the 87,430 CAP patients, 91,189 laboratory tests were conducted, including 13,703 cases of *S. pneumoniae*. Among all the studies included, the proportions of CAP caused by *S. pneumoniae* ranged from 0.7 to 65.1%. Figure 3 presents the unadjusted, study-specific proportions of *S. pneumoniae* as the causative agent for CAP over the study duration, with 95% CI. The pooled prevalence of *S. pneumoniae* was 14.8% (95% CI: 12.3–17.8%). In studies in which bacterial culture constituted the sole detection method, the pooled prevalence decreased from 16.5% (95% CI: 15.0–18.2%) in the period 1996–2000 to 8.4% (95% CI: 6.3–11.0%) in the period 2016–2020 (*P* < 0.001). A similar trend was also observed in studies that employed a combination of bacterial culture and UAT, from 17.4% (95% CI: 16.3–18.7%) in 1996–2000 to 13.5% (95% CI: 10.7–16.8%) in 2016–2020 (*P* < 0.001).

### Subgroup analysis and univariate and multivariate meta-regression

By univariate meta-regression of study characteristics, we found no statistically significant difference in the observed prevalence of *S. pneumoniae* by continent (*P* = 0.060), study design (*P* = 0.051), or clinical setting (*P* = 0.099), whereas a significant difference by country (*P* < 0.001), developmental stage (*P* = 0.010), or laboratory diagnostic method (*P* = 0.031) was observed (Appendix Table 2). In the subgroup analysis, the heterogeneity between each subgroup was relatively high. The prevalence of *S. pneumoniae* was higher in England, Spain, and the Netherlands, at 26.2%, 25.4%, and 24.6%, respectively, whereas Thailand (3.1%), Poland (3.9%), and Israel (5.9%) presented lower prevalence rates. The prevalence of *S. pneumoniae* was greater in developed countries than in developing countries (16.1% vs. 8.5%, *P* = 0.010). The prevalence of *S. pneumoniae* was greater with PCR and the combination of multiple detection methods (21.1% and 19.2%, respectively), whereas only culture (9.2%) and only UAT (12.9%) had lower prevalence rates. Multivariate meta-regression revealed that the adjusted *R²* of the regression model formed by these covariates was 31.1%, with a *P* value of 0.002.

### Serotype

**Fig. 4:**
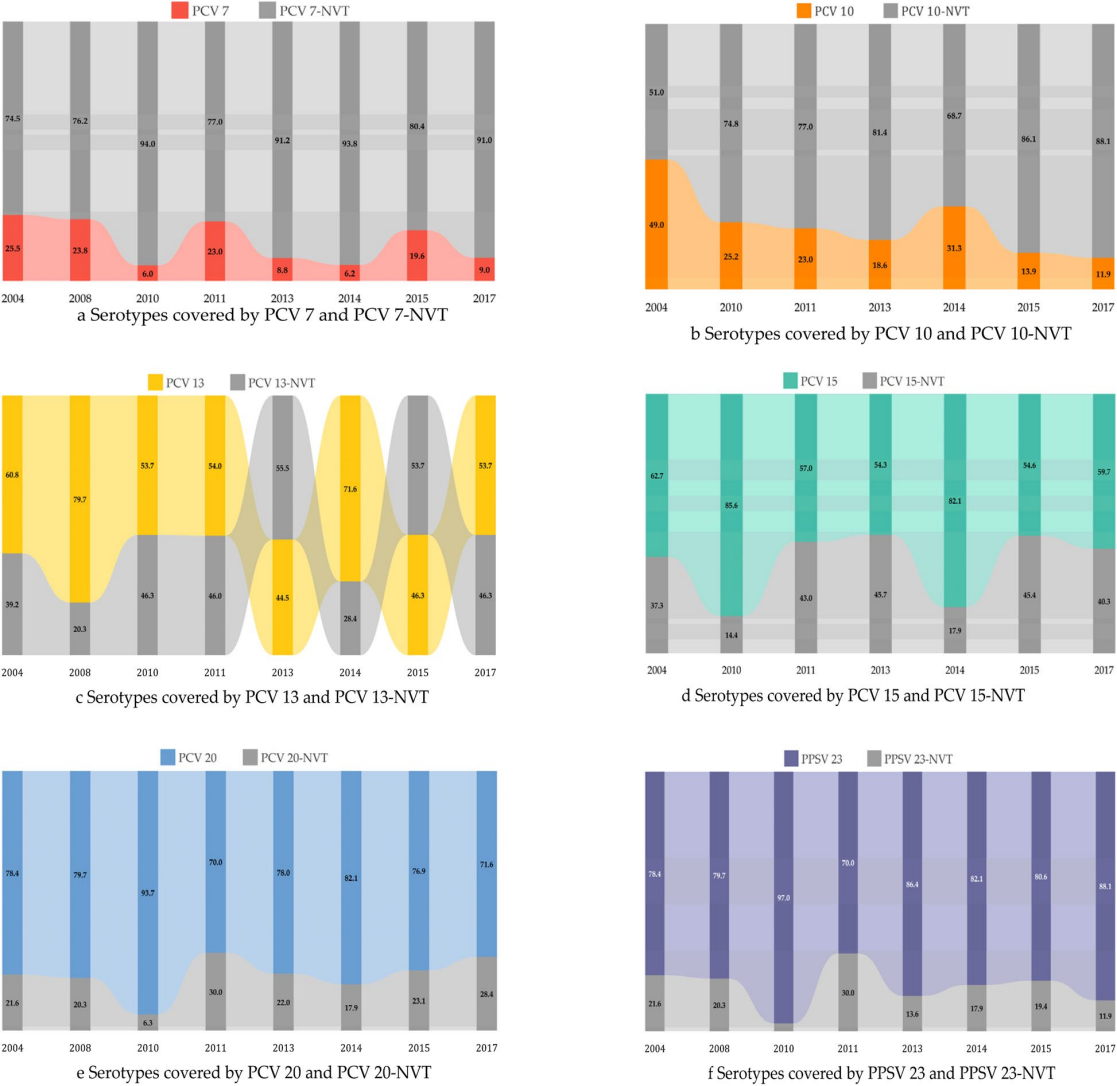
(**a**-**f**). Distribution of pneumococcal vaccine serotypes, with the abscissa representing the initial year of the study

**Fig. 5:**
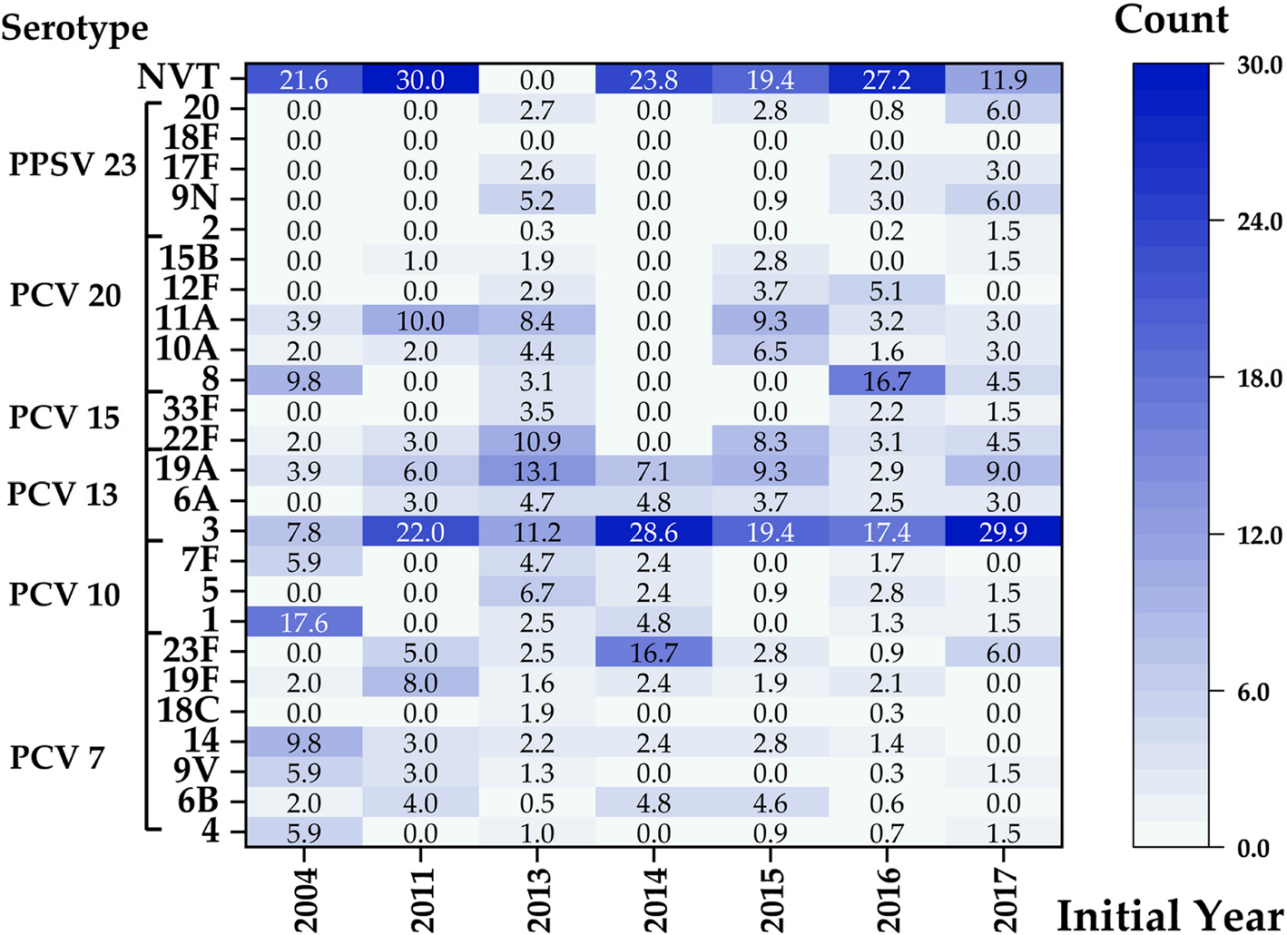
Serotype distribution of *S. pneumoniae* in elderly patients with pCAP. (6 A and 18 C are not included in PPSV 23.)

A total of eleven publications reported data pertaining to the serotypes involved in elderly patients with pCAP. We analysed the coverage of the six known pneumococcal vaccine serotypes to determine whether there was a change from 2004 to 2017 (Fig. 4). The coverage increased in correlation with the inclusion of additional serotypes in the vaccine. Overall, the PCV 7-serotype coverage was 9.6%, whereas the coverage was 19.4% for PCV 10, 53.5% for PCV 13, 60.5% for PCV 15, 85.2% for PCV 20, and 88.6% for PPSV 23. We further analysed the changes in serotype coverage for each vaccine over time. The findings presented a gradual decrease in the proportion of serotypes covered by PCV 7, from 25.5% in 2004 to 9.0% in 2017. Similarly, the coverage of PCV 10 decreased from 49.0 to 11.9%, whereas that of PCV 13 decreased from 60.8 to 53.7%. In contrast, no significant downwards trend in serotype coverage was observed for the other three vaccines: 62.7% vs. 59.7% for PCV 15, 78.4% vs. 71.6% for PCV 20, and 78.4% vs. 88.1% for PPSV 23. Among the 2302 pneumococcal isolates identified in seven publications, the most prevalent were 3, 8, 19 A, 22 F, 11 A, 5, 9 N, 12 F, 6 A, and 10 A, which account for 18.0% (414/2302), 10.5% (241/2302), 9.3% (213/2302), 7.7% (178/2302), 6.9% (159/2302), 5.0% (114/2302), 4.3% (100/2302), 4.3% (98/2302), 4.1% (94/2302) and 3.6% (82/2302), respectively. The prevalence of serotypes 7 F, 1, 14, 9 V, 6B and 4, which are covered by PCV 7 and PCV 10, decreased to negligible levels from 2014 to 2022. Conversely, the prevalence of the additional serotypes in higher-valence vaccines has increased, with serotype 3 showing the most significant increase (Fig. 5).

## Discussion

Over the past two decades, three meta-studies have been conducted to examine the prevalence of pneumococcal pneumonia in Europe [11], India [5] and globally [6]. The findings indicated that the global prevalence of pneumococcal pneumonia was 27.3% in adults prior to 2010, as determined by UAT and bacterial culture [6]. Additionally, the prevalence was approximately 19% in Europe and India, which may be constrained by the geographical scope or the sensitivity of the detection method [5, 11]. These meta-analyses did not analyse the distribution of pneumococcal serotypes. In view of the potential for temporal and immunization-programmatic changes in the disease burden of *S. pneumoniae*, a search was conducted for studies on pCAP in older patients published between 2013 and 2023. This study was performed to estimate the proportion of CAP caused by *S. pneumoniae* among elderly individuals worldwide. To obtain a more reliable distribution of *S. pneumoniae*, outpatients or inpatients from 19 countries who were diagnosed with CAP by radiographic imaging were included in the study. The laboratory diagnostic methods employed in the present study included not only bacterial culture and UAT, but also the more sensitive method of PCR. The results demonstrated that approximately 17.0% of CAP cases were caused by *S. pneumoniae*, which was lower than that reported by Said, M.A. in 2013 [6]. PCV has been shown to reduce the incidence of CAP and is not only effective in vaccinated children but also protects unvaccinated adults through herd immunity effects [62]. Following the introduction of PCV 7 in 2000 and PCV 10 and PCV 13 in 2009, an increasing number of countries are incorporating pneumococcal vaccines into their national immunization programmes. The global coverage of the final dose of PCV was 65% in children in 2023 [63]. It can thus be inferred that the preceding results imply a reduction in the overall burden of pneumococcal disease over the decade, which is likely a consequence of large-scale vaccination in the population, including children and elderly individuals.

The distribution of pneumococcal serotypes represents a significant outcome of this study. The implementation of a vaccination programme has the potential to prevent the occurrence of pneumococcal pneumonia. The impact of the intervention can be evaluated qualitatively by comparing the proportion of disease caused by different pneumococcal serotypes over time. The findings of this study indicate a notable decrease in the coverage of earlier licenced vaccine serotypes over the past 14 years, particularly in the case of PCV 7. The binding of pneumococcal polysaccharides to immunogenic protein carriers has been demonstrated to increase the antibody response and induce immune memory, resulting in direct and indirect protection that has been demonstrated to significantly diminish the associated disease burden. Accordingly, the extensive deployment of PCV 7 has prompted shifts in the major serotypes within the population [47, 64], as presented in this study. Notably, irrespective of geographical location, the predominant serotypes associated with pCAP remain those that are protected by the new PCV vaccine, specifically serotypes 3, 8, 19 A, 22 F and 11 A. Some studies [65–67] indicate that the immunogenicity of vaccines against serotype 3 is not as robust as that against other serotypes. This finding is believed to be related to the changing clade of serotype 3, which may impact the protective efficacy of the population. Consequently, the prevalence of *S. pneumoniae* in serotype 3 within the population has not been effectively suppressed. The present study did not identify any alterations in the coverage of additional serotypes incorporated into PCV 15 or PCV 20, including 22 F, 8, and 11 A. This is attributed to the relatively brief period during which these vaccines have been commercially available, which has precluded any meaningful evaluation of their impact. Importantly, future studies should concentrate on the impact of these products on the prevalence of disease and the evolution of pneumococcal serotypes in a variety of geographical and epidemiological settings. In certain regions, individuals aged 65 years and above have been included in complimentary vaccination programmes. Nevertheless, the findings of this study indicate that the level of coverage for pneumococcal vaccination is less than 50% in numerous countries and regions. Consequently, there is a clear need for increased promotion of the vaccine to effectively reduce the burden of pneumococcal pneumonia. Furthermore, the research and development process needs to be accelerated to increase the immunogenicity of serotype 3.

This study was drawn primarily from Europe, North America and Asia. Discrepancies were observed in the reported prevalence rates across different countries. We tried to obtain as much information about the included studies as possible to explain these differences. Through meta-regression analysis and the inclusion of more recent studies, we were able to confirm significant differences that were independent of covariates. We accounted for the influence of various covariates, such as different countries, developmental statuses, study designs, health-care settings, laboratory diagnostic methods, average study durations, and average ages of patients, on the observed prevalence of *S. pneumoniae*. The model accounted for 31.1% of the heterogeneity. This correction revealed that unobserved variation remained among studies regarding the observed proportion of *S. pneumoniae* in CAP, such as bacterial resistance and vaccination. Notably, the study revealed a lower prevalence rate in developing countries than in developed countries, such as England, Spain, and the Netherlands. This finding may be attributable to the timing of the study and the testing methods used. The studies from developed countries included in this review commenced as early as 1996, whereas the majority of the studies in developing countries were conducted after 2009. A significant proportion of the literature in developed countries originated from earlier studies of pneumococcal pneumonia conducted prior to the introduction and widespread adoption of pneumococcal vaccines. Furthermore, more sensitive tests, such as PCR and urine antigen detection, are more prevalent in developed countries, whereas bacterial culture methods are predominantly employed in developing countries. Diagnostic and registration methods frequently suggest to be inadequate as a result of a dearth of requisite medical resources, resulting in numerous cases of respiratory infections being undiagnosed in the laboratory. Therefore, the potential for underreporting and underestimation of pneumococcal disease may result in an invalid estimation of disease incidence, consequently resulting in a significant underestimation of the true burden of pneumococcal CAP in developing countries [68].

Our study revealed that over time, pneumococcal studies have undergone a transition from relying primarily on bacterial culture as a detection method to an increasing number of studies using PCR testing. The use of PCR for the detection of *S. pneumoniae* is both sensitive and specific compared with the traditional method of culture [69, 70]. In particular, PCR is an effective means of identifying cases in which the culture has yielded a negative result. Consequently, the detection rate of *S. pneumoniae* was also markedly higher in studies that performed PCR assays more frequently than in those that performed all other diagnostic tests. Furthermore, the sensitivity of the UAT assay was greater than that of the bacterial culture method. This result is consistent with the findings of a previous study [57] in which blood cultures, sputum cultures and urine antigen tests were performed simultaneously in patients with CAP. As previously stated by Ghia, C. J. [5], the prevalence of *S. pneumoniae* as a cause of CAP has been underestimated because of the lack of sensitivity of the technique for the isolation of *S. pneumoniae* from blood. Currently, many countries have used ssUAD to detect pneumococcal serotypes, which can identify 24 serotypes included in pneumococcal vaccines. However, ssUAD works only for bacteremic CAP [71, 72]. The burden of *S. pneumoniae* may be underestimated by the ssUAD assay because nonbacteremic CAP is more common in CAP [6, 32, 73, 74]. From a public health standpoint, long-term surveillance employing more sensitive diagnostic techniques may be crucial. Furthermore, it is imperative that patients with CAP be screened more frequently to reduce the incidence of misdiagnosis and inadequate treatment. As the availability of these methods increases and their use in CAP improves, it is likely that the underdiagnosis of CAP will be reduced and that more reliable data on the burden of pneumococcal disease in older adults will be available.

This study has several limitations. The dearth of data from studies conducted in Africa and low-income countries has resulted in an inadequate understanding of the prevalence of CAP caused by pneumococcus in these regions. Therefore, the global prevalence of pCAP in elderly individuals may be underestimated. Furthermore, additional factors such as antibiotic treatment [43, 51, 61], immunosuppression [24], comorbidities [15], a history of smoking [55], and a history of respiratory disease [39] may also influence the proportion of *S. pneumoniae* detected. Despite our best efforts to collect as much data as possible and to use more comprehensive inclusion and exclusion criteria to correct for the effects of these factors, the lack of detailed information in the literature, as previously mentioned, resulted in incomplete identification of heterogeneity in the included studies. In addition, not all studies provided explicit information on pneumococcal vaccine coverage, thus precluding the incorporation of vaccination into the model to explore the association between *S. pneumoniae* prevalence and vaccination rates in greater depth.

## Conclusion

Our study offers a comprehensive overview of the global prevalence of *S. pneumoniae* from a broader perspective, indicating a notable decrease in the overall burden of pneumococcal CAP (pCAP). The variability in prevalence was found to be associated with many factors, including the stage of social development, laboratory diagnostic methods and vaccination programmes. The main serotypes associated with pCAP remain those protected by PPSV and PCVs, which were recently introduced. These findings lend further support to the assertion that pneumococcal vaccines are relevant in elderly individuals. In developing countries, more research is needed to clarify the burden of disease and vaccine effectiveness to inform local policy recommendations on pneumococcal vaccination in older adults.

## Data Availability

The datasets used and/or analysed during the current study are available from the corresponding author on reasonable request.

## Appendix

**Table 2:** Pooled S. *pneumoniae* detection rates overall and in subgroups by study characteristics

Population	Data (*n*)	Total (*n*)	Positive (*n*)	Proportion (95% CI)(%)	I² (%)	*P* for interaction
**Continent**	65	91,189	13,703	14.8 (12.3, 17.8)	98.3	0.060
Asia	36	25,635	3,333	12.5 (9.6, 16.1)	95.4	
Europe	21	25,508	5,505	20.0 (14.7, 26.7)	98.8	
North America	78	40,046	4,865	13.7 (10.6, 17.5)	88.0	
**Country**	65	91,189	13,703	14.8 (12.3, 17.8)	98.3	< 0.001
America	4	12,656	1,570	16.1 (8.7, 27.9)	90.1	
Canada	4	27,390	3,295	12.3 (11.1, 13.6)	88.8	
China	2	1,009	70	7.2 (5.2, 9.9)	40.8	
England	7	7,879	1,872	26.2 (13.0, 45.7)	99.4	
Iceland	2	665	106	15.8 (11.9, 20.8)	59.4	
Israel	2	996	65	5.9 (2.1, 15.6)	93.1	
Japan	13	8,648	1,380	18.0 (10.5, 28.9)	96.0	
Korea	9	12,217	1,441	11.6 (9.5, 14.0)	91.1	
Malaysia	1	89	7	7.9 (3.8, 15.6)	-	
Netherlands	1	505	124	24.6 (21.0, 28.5)	-	
Norway	2	529	45	8.5 (6.4, 11.2)	0	
Pakistan	2	698	103	10.8 (3.4, 29.5)	93.2	
Poland	1	51	2	3.9 (1.0, 14.4)	-	
Portugal	1	1,635	192	11.7 (10.3, 13.4)	-	
Singapore	3	300	54	15.3 (5.1, 38.0)	90.2	
Spain	6	13,762	3,099	25.4 (19.1, 33.0)	97.9	
Thailand	1	490	15	3.1 (1.9, 5.0)	-	
Turkey	3	1,218	198	10.5 (3.4, 28.0)	93.2	
Greece	1	482	65	13.5 (10.7, 16.8)	-	
**Developmental stage**	65	91,189	13,703	14.8 (12.3, 17.8)	98.3	0.010
Developing country	9	3,504	393	8.5 (5.3, 13.4)	94.2	
Developed country	56	87,685	13,310	16.1 (13.3, 19.4)	98.5	
**Study design**	65	91,189	13,703	14.8 (12.3, 17.8)	98.3	0.051
Cohort study	39	46,250	7,898	16.9 (13.2, 21.5)	98.6	
Cross-sectional study	22	15,235	2,258	12.1(8.9, 16.3)	94.1	
Surveillance	4	29,704	3,547	11.8 (10.0, 13.8)	92.6	
**Clinical setting**	65	91,189	13,703	14.8 (12.3, 17.8)	98.3	0.099
Outpatient or emergency	6	7,449	790	8.9 (5.4, 14.6)	97.7	
Inpatient	53	82,769	12,694	15.8 (13.0, 19.2)	98.5	
ICU	6	971	219	12.9 (5.5, 27.3)	93.2	
**Laboratory diagnosis**	65	91,189	13,703	14.8 (12.3, 17.8)	98.3	0.031
Culture	18	11,986	1,317	9.2 (5.8, 14.2)	97.1	
UAT	8	8,171	1,232	12.8 (9.6, 17.0)	95.4	
Culture and UAT	10	37,495	5,904	17.0 (13.2, 21.6)	99.0	
PCR	3	1,581	232	21.1 (5.2, 56.4)	98.2	
Mixed	26	31,956	5,018	19.2 (14.8, 24.5)	98.8	

mixed: combination of multiple detection methods
